# The Conservation of Native Domestic Animal Breeds in Nordic Countries: From Genetic Resources to Cultural Heritage and Good Governance

**DOI:** 10.3390/ani11092730

**Published:** 2021-09-18

**Authors:** Ulla Ovaska, Auli Bläuer, Charlotte Kroløkke, Maria Kjetså, Juha Kantanen, Mervi Honkatukia

**Affiliations:** 1 Natural Resources Institute Finland (Luke), Latokartanonkaari 9, 00790 Helsinki, Finland; ulla.ovaska@luke.fi; 2 School of History, Culture and Arts Studies, University of Turku, 20014 Turku, Finland; aultou@utu.fi; 3 Department for the Study of Culture, University of Southern Denmark, 5230 Odense, Denmark; charlottekro@sdu.dk; 4 Nordic Genetic Resource Center, NMBU, P.O. Box 5003, 1432 Ås, Norway; maria.kjetsa@nordgen.org; 5 Natural Resources Institute Finland (Luke), Myllytie 1, 31600 Jokioinen, Finland; juha.kantanen@luke.fi

**Keywords:** native breeds, genetic resources, conservation genetics, cultural value, governance, conservation programme

## Abstract

**Simple Summary:**

Native breeds are domestic animals that have adapted to local conditions. Their genetic and cultural values are high. The conservation of these breeds is maintained by national conservation programmes and agricultural support schemes in Nordic countries. In addition to financial support, the conservation of native breeds requires that their importance in society be widely understood and recognised. This is especially crucial in the local communities in which such breeds are kept. Farmers raising native breeds should be highly motivated to utilise these breeds in animal production. This article examines the extent to which farmers and stakeholders recognise the genetic and cultural significance of conservation, and how the requirements of good governance are met in current conservation arrangements. Moreover, we contemplate the potential to amalgamate the management of animal genetic resources and their cultural environment.

**Abstract:**

Native breeds are domestic animal populations that have adapted to their habitats. The genetic value of breeds has been known for a long time, and recently more attention has been paid to their cultural value. Due to both ecological and cultural significance, it is important that native breeds continue to be bred in their native environments. This is supported by various financial support schemes. Support schemes rarely cover the financial gap in output compared to commercial breeds. A solution to this has been sought in special products, such as cheese or wool, and other businesses, such as animal-assisted care and tourism. Less attention has been paid to the role of administration and good governance in the maintenance of native breeds. In this study, a questionnaire was sent to all registered keepers of native breeds in Finland. This survey clarified their reasons for keeping native breeds and their ideas for improving governance structures and practices. The results were discussed in stakeholder workshops, and in a Nordic context. The results show that genetic and cultural values are recognised in several documents and programmes, but farmers need to be engaged more in the design of support schemes and practices.

## 1. Introduction

Native breeds are farm animal populations that have adapted to local conditions, including traditional agricultural production systems and environments. They originate from certain geographical regions, are adapted to these regions’ environmental conditions, and are often utilised therein [1]. Native breeds are less influenced by modern breeding techniques; rather, their selection is based on adaptability, and other important traits linked to local traditions and animal husbandry practices, such as meat and milk yield, or draught power [2].

During the past few decades, the number of native breeds has diminished due to the requirements of intensive livestock farming and global economic development. It is estimated that nearly 30% of native breeds worldwide are endangered [3]. Today, the value of indigenous breeds—from genetic conservation to socioeconomic and cultural heritage viewpoints—is fairly well known (e.g., [4]). In particular, their genetic and cultural values are widely recognised by the scientific community, stakeholders (such as farmers), political decision-makers, lecturers of livestock production, and the wider public [5,6,7]. Alongside traditional roles in food supply and security, new rural livelihoods have developed for native breeds in various services, such as animal care and tourism, reintroduction to traditional grazing, and special niche products (e.g., handicrafts and delicacies) [8]. 

Despite the numerous values of farm animal breeds (e.g., sustainability and culture), economic value is often prioritised over socioeconomic importance or cultural heritage [9,10]. Quantifying and evaluating these nonmaterial cultural values is challenging, because they are often intangible, and typically not recognised by markets [10,11]. 

Genetic diversity generally underpins population resilience; its reduction will eventually lead to the extinction of the breed or species. In general, genetic diversity can be viewed at many levels, such as the diversity of individuals (i.e., genetic differences between individuals) within a population, the differences between populations, and the level of the ecosystem. The effectiveness of conservation efforts to maintain genetic diversity can easily be measured with genetic tools, or by using pedigree record data, as trends in genetic diversity over various timespans. Determining the conservation value of genetic material is important in order to assess the priorities and management of genetic diversity conservation.

While native breeds are frequently valued as genetic resources for future animal breeding and economic importance, they also have cultural value. Cultural anthropologists and critical animal scholars stress that understanding human and nonhuman animal relations is vital in the production of culture. For example, the native Northern Finncattle, Swedish Mountain Cattle, and Coloursided Troender and Nordland Cattle (STN), from Finland, Sweden, and Norway, respectively—collectively termed Nordic mountain cattle breeds—gain value when, in interviews with animal owners, they emerge as “friends”, “family members”, “human companions” and “colleagues” [12]. Moreover, when long-lost cultural traditions become re-established, such as in the reintroduction of these cattle breeds into the northern regions, Nordic cattle help to restore Nordic cultural heritage. Thus, the value of native cattle goes beyond their genetic value, and extends to how they symbolically and materially connect with humans and regional environments. 

The prehistory and history of native breeds are important parameters for their cultural value and conservation. The genetic variation and structure of domestic animal breeds have been affected by human migrations, environmental constraints, and animal husbandry practices and preferences. Thus, archaeological and zooarchaeological materials may be utilised in understanding temporal changes in genetic diversity, setting conservation priorities, and developing conservation strategies (e.g., [13,14]). Traditions and shared history with humans are important components of the cultural value of native animal breeds.

The governance of genetic resources has a matrix-like structure. Conservation measures take place at multiple levels, with the vertical and horizontal interplay among stakeholder institutions. A comprehensive understanding of the values connected to native breeds and their genetic resources is needed in order to manage their conservation effectively. This implies information on a breed’s characteristics, regular monitoring of population size and structure, and genetic diversity within and between other breeds as indications of uniqueness. Information on geographical distribution and production environments is also needed. Knowledge of the importance of socioeconomic, cultural, historical, and economic value is also essential in management (e.g., [15]). 

In addition to the above-mentioned factors, prosperous governance is important in the successful conservation of biodiversity and the reintroduction of cultural heritage [16,17]. Governance implies how policies, institutions, and processes shape decision making, and how decisions are made in conservation planning and management [18]. This involves networks and linkages across various levels and sectors [16]. Conservation governance starts from UN international agreements, continues to the EU and national levels, and goes further to the local society level. This involves, for example, the rural, agricultural, and environmental sectors [17]. Good governance can be defined in different ways, but the concept includes at least the elements of legitimacy, transparency, accountability, inclusiveness, fairness, integration, capability, and adaptability [19]. Ideally, people have the right to co-create decisions that affect their lives and livelihoods. 

At present, the conservation of native breeds is maintained through conservation programmes, and by subsidy policies intended for farmers in Nordic countries [20,21,22,23,24]. Living populations are maintained (in vivo conservation) and genetic materials (semen and embryos) are stored in a cryobank (in vitro conservation). Since it is a question of interplay between different levels and sectors of governance, the arrangements ideally promote both conservation and local development. This is typically done by providing economic incentives for conservation and related livelihoods. 

This article explores how information gathered from different sources can be used to enhance the conservation of biological and cultural heritage diversity in the Nordic countries, and to promote good governance. The main questions are (1) how effective the conservation is from genetic viewpoints, (2) to what extent farmers and other stakeholders recognise the genetic and cultural value of native breeds, and (3) how the requirements of good governance are met. Furthermore, we discuss (4) experience acquired from Nordic approaches to the management of conservation programmes, and (5) how cross-border cooperation can be harnessed in the management of transboundary breeds (i.e., breeds that are found in several countries). 

We examined these questions based on a survey targeted at breeders of native farm animal breeds in Finland, conclusions of stakeholder workshops organised in 2018 in Finland (see [25]), experience gained from an ongoing project aiming to increase awareness of the traditions and history related to Nordic mountain cattle breeds [26], and reviewing Nordic countries’ national strategies [20,21,22,23,24]. 

## 2. Materials and Methods

The main aim of this study was to develop the conservation of native farm animal breeds. This focus was considered in tailoring methods, selecting relevant stakeholders, and deciding on the appropriate level of their engagement [27]. The study was conducted using stakeholder analysis methods [28]. During the current EU programme period (2014–2020), there have been no studies elaborating the perspectives of farmers or other stakeholders in Finland. Even those published during the period collected their data during previous programme periods (e.g., [7,8,17,29,30,31]). Therefore, the stakeholder analysis was needed in order to gather information about the perspectives and current issues relevant to farmers and other stakeholders. 

Reed et al. [28] distinguish between normative, instrumental, and descriptive rationale of stakeholder analyses. While normative analysis is used for legitimation, and is instrumental for achieving desired outcomes, we chose descriptive stakeholder analysis due to the need for more understanding of the current state of affairs. The starting point of the study was the current conservation system, and the system boundaries that it created. We continued gathering information from stakeholders with a combination of questionnaire, workshop, and “snowball sampling” methods [28]—that is, our knowledge of important questions and relevant stakeholders supplemented during the research process. In addition to keepers of native farm animal breeds, we identified stakeholders active with each native farm animal breed on different governance levels—from NGOs to ministries, and from research to private business. In addition to data obtained from the survey, this was done with information gathered from previous research and current conservation programmes (e.g., [7,8,17,21]). We then categorized the stakeholders and identified relationships between them in order to understand the governance of conservation. Finally, we reflected the obtained conclusions in the implementations of the Nordic conservation systems. In this research process, we followed the stakeholder analysis methods presented by Reed et al. [28]. 

In order to obtain a comprehensive picture of conservation at the implementation level, we constructed a questionnaire, with the possibility of open answers, for the breeders of native farm animal breeds in Finland. The information obtained from the survey was used in three consecutive workshops with selected stakeholders that represented different governance levels. Finally, the results from the survey and workshops were discussed with the members of the Advisory Board for the Genetic Resources of the Finnish Ministry of Agriculture and Forestry.

**Figure 1:**
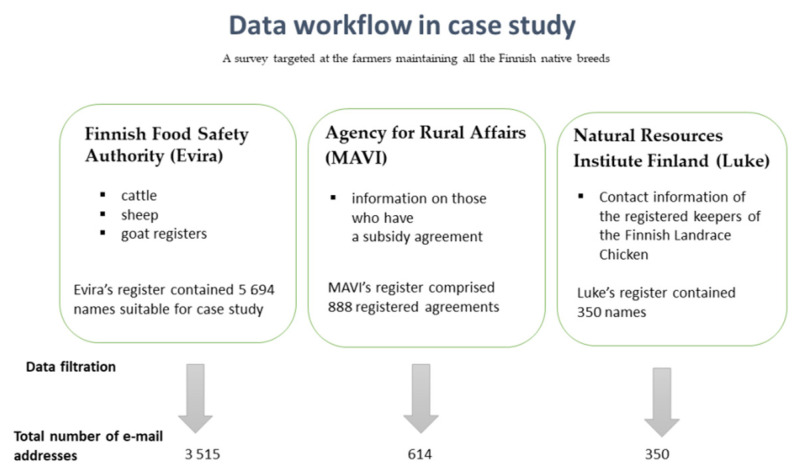
Data workflow in the case study. The figure demonstrates the utilisation of multiple registers to comprehensively reach the stakeholders keeping indigenous breeds. Notice that the Finnish Food Safety Authority (Evira) and Agency for Rural Affairs (MAVI) merged in 2019 and formed the current Finnish Food Authority.

Thus, the case study consisted first of a survey targeted at the farmers maintaining all of the Finnish native breeds. A request to obtain the contact information for the correct target group was sent to all databases that maintain native-breed information: the Finnish Food Safety Authority (Evira, currently the Finnish Food Authority), for cattle, sheep, and goat registry information; Natural Resource Institute Finland (Luke), for the Finnish Landrace Chicken; and the Agency for Rural Affairs (MAVI, currently the Finnish Food Authority; the previous Evira and MAVI were merged in 2019), for information on those who had signed a subsidy agreement (Figure 1). The latter register is for farmers with subsidy agreements since, in addition to regular agricultural support payments, support can be obtained by concluding an environmental contract with the government. 

According to the subsidy agreement, the farmer engages in keeping certain native-breed animals, following the requirements of the contract. According to the breeding requirements defined in the agreement, an eligible animal must produce a certain number of purebred offspring during the contract period. The other stipulation concerns the length of the contract period. 

The breeds in the survey were Finnsheep, Åland Sheep, Kainuu Grey Sheep, Finngoat, Eastern Finncattle, Western Finncattle, Northern Finncattle, Finnhorse, and the Finnish Landrace Chicken. The first register provided 5694 names, and the latter registered 888 agreements. It is worth noting that, in some cases, there were several animal species and breeds on the same farm; therefore, the total number of e-mail addresses was 3515. Similarly, out of the 888 agreements, there were several agreements on the same farm; thus, 614 e-mail addresses were received. 

A Webropol questionnaire (see Appendix A) was sent to the e-mail addresses in early August 2018, and a reminder was sent to all e-mail addresses in late August 2018. A total of 517 responses were received, so the response rate was ~15%. The questionnaire contained seven multiple-choice questions, with a section in which the research participants could write their thoughts via free word. The questions included which breeds the respondents keep, how, and why; what challenges there are in the current system; what development needs there are in the current system; and how the respondents would organize the conservation. The survey also aimed to identify current issues and topics at the implementation level. Thus, the aim of the survey was not to conduct a statistical study, but to obtain information so as to continue the research process [27,28].

In the second phase, research data were collected in three consecutive virtual stakeholder workshops held in September 2018. The stakeholder workshops gathered 15 participants engaged in the conservation of native breeds. The participants represented breeding organisations, agriculture advisory agencies, research organisations, and public farm herds—which are so-called “living gene banks” for native cattle and sheep breeds—as well as private enterprises. The results of the survey and the workshops were discussed with the Advisory Board for the Genetic Resources of the Finnish Ministry of Agriculture and Forestry in December 2018. 

For this paper, the results of the survey, workshops, and discussion were analysed regarding the following three factors:

(1) How efficient is conservation from a genetic viewpoint?
(2) How well are genetic and cultural values recognised?
(3) To what extent are the elements of good governance met?

Third, we analysed Nordic conservation programmes (Denmark, Finland, Iceland, Norway, and Sweden), including their subsidy policies, to understand the main similarities and differences in conservation strategies between the Nordic countries. 

In the last part of this paper, we reflect on the concept of the 3MC Nordic Mountain Cattle project [26] as a fresh practice by which to administer a conservation programme. This ongoing project is designed to understand the importance of the concurrent maintenance of cultural inheritance and the biodiversity of animal genetic resources and their environments; it is an interdisciplinary venture that studies the history, cultural heritage, and modern and archaeological genetics of three transboundary cattle breeds: Northern Finncattle, Swedish Mountain Cattle, and Coloursided Troender and Nordland Cattle (STN), in Finland, Sweden, and Norway, respectively. The experiences of common history and shared ancestors are utilised when assessing a holistic conservation programme for the genetic resources and cultural heritage of Nordic transboundary cattle breeds. 

## 3. Results 

### 3.1. How Efficient Is Conservation from a Genetics Viewpoint, and How Well Is Genetic Value Recognised?

#### 3.1.1. Farmer Perspectives

Among the respondents of the survey, 48% were committed to preserving native breeds through the official subsidy system. Most responses were received from cattle breeders (50.4%) and sheep keepers (33.7%), but farmers keeping goats (2.1%), chickens (5.4%), and horses (8.3%) also answered the questionnaire (see Table 1). Of the respondents, 153 answered about keeping two or more native breeds. The maximum number of different breeds per farmer was five. Thirteen farmers announced that they would quit keeping native breeds.

**Figure 2:**
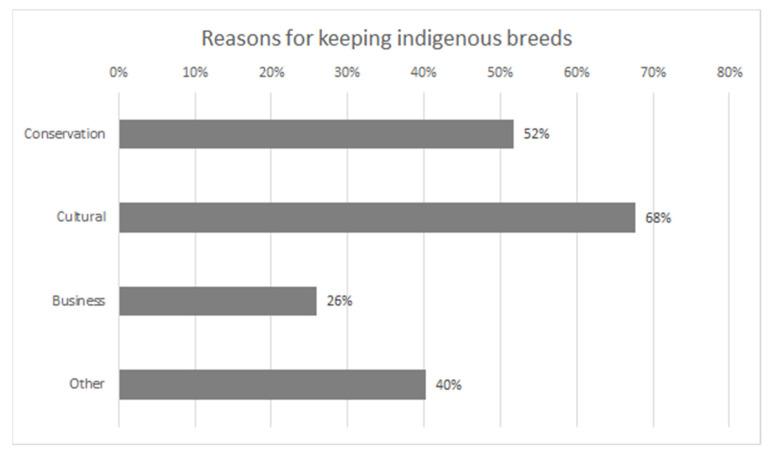
Farmers’ reasons for keeping indigenous breeds. Percentage of respondents for each answer. Multiple answers per respondent possible.

The conservation of genetic resources was the motivation for 52% of the respondents (Figure 2). For questions involving respondents writing their answers, characteristics of the breeds were mentioned as reasons for keeping them: *“Good grazers, good maternal characteristics.”; “Toughness, smaller size is feasible to* [our] *old barn.”; “Breed characteristics, good grazer, hornless, easy calving.”; “Smart, modest, healthy”; “Fertile”*. Previous research has also shown that the genetic resources and breed characteristics are important for the keepers of native farm animal breeds [29].

Keeping native breeds was seen as being connected to landscape management, and as a logical option for the conservation of ecosystems: *“[Maintenance of] biodiversity”; “Management of fields as ecologically as possible”; “Very suitable for natural pastures and yet possible to produce meat as well”*. The production of different ecosystem services is often underlined as a reason to keep the native farm animal breeds, partly due to their lower yield compared to other breeds [17].

Farmers keeping the native breeds for basic meat, milk or wool production commented briefly: *“They are in production”*. The respondents, as an important reason for keeping native breeds, cited their ability to adapt to a modest environment. Some stated keeping them out of commented that the reason was the agricultural subsidy scheme intended for native breeds. According to a previous survey of native cattle and sheep breeders in Finland, economic reasons have not sparked the interest in keeping the breeds [29]. However, the economic incentives were important to continue keeping the native farm animal breeds. In a study on native farm animal breeds across Europe, the first reason was productivity, followed by tradition [30].

Many expressed concern that the lack of financial resources from the government hindered preservation agreements. Another concern was that some native breeds seemed to have taken precedence, and the support system was skewed in favour of certain breeds. There were also concerns that, due to this bias, meat- and milk-producing cattle breeds were made into suckler breeds to maximise the number of agreements and payments, which would harm the conservation results.

Every third respondent expressed an interest in committing to a specific environmental agreement aimed at protecting traditional biotopes and landscapes. On the other hand, the scarcity of subsidies reduces the motivation to keep native breeds for landscape management.

#### 3.1.2. Stakeholder Perspectives

The main goal of the stakeholder sessions was to elaborate on the findings from the farmers’ survey. It was mentioned that support may need to be targeted at farmers, who are largely responsible for conserving genetic diversity, and whose animals are provided for research and breeding. The importance of securing funding for the few living gene banks was emphasised. Furthermore, quotas were proposed for different breeds in the environmental agreements in order to keep the number of breeds in balance. It is essential to acknowledge the diversity among native farm animal breeders in order to develop and implement conservation policies and programmes [31].

### 3.2. How Well Is Cultural Value Recognised?

#### 3.2.1. Farmers’ Perspectives

The percentage of respondents who keep breeds for cultural reasons was 68%. These reasons included history and tradition: *“Since 1898, as a production breed”*. For some, intergenerational aspects were future-oriented: *“Easily recognizable own animals for grandchildren”*. Similar results have been obtained in previous research [29].

As illustrated in the ongoing 3MC Nordic Mountain Cattle project [26], farm animals play an important role in maintaining cultural heritage, including intergenerational ties. Moreover, the rarity, originality and locality of the breeds were other important factors in keeping them: *“They are ‘peculiar’ and domestic”*. According to the experience from the above-mentioned project, “domestic” cattle are achieving additional value as national symbols. Native farm animal breeds have been identified as national symbols in previous research, e.g., the Finnhorse for its role in the wars, or that of Finncattle in self-sufficient rural communities in the past [7].

There were other individual reasons or preferences for choosing native breeds: *“I love them”; “Own interest and hobby and as Pets”*. In describing mountain cattle in such affectionate terms (e.g., “I love them”) and in their capacity as pets, reflections of farm animals emerge outside the category of “livestock”, and instead take on the position of companion animals. This extends to animal aesthetics when, for example, Northern Finncattle are described as *“beautiful to look at”*. In taking on these positions as pets and aesthetically pleasing nonhuman animals, farmers address concerns related to animal rights: *“So that at least one animal may live a free life the way it wants”*; and individualising mountain cattle when describing them as *“There’s some rebel-spirit involved”*.

Although cast as companion animals, farm animals are, in the material sense, also valued for the products they provide, which are closely aligned with local gastronomy and clothing culture: *“Excellent healthy milk, cheese and ‘marbled’ meat”; “[…] traditional handicrafts”*, and of their local community: *“All people and school children in our village love them and want to pet them”; “Job for our youngest, disabled child”*. Thus, the animals’ economic value is not only about food and fibre, but also gastronomy and clothing [7].

#### 3.2.2. Stakeholder Perspectives

Stakeholder workshops sought alternatives and complementary measures to the agricultural subsidy system in order to increase the value of genetic resources, such as the branding and labelling of native-breed products. Furthermore, some argued that the utilisation of a short food supply chain from farmer to consumer with fewer intermediaries—such as local direct trade, farmers markets, or shops—could be used to increase the profitability and popularity of native breeds in food production. The importance of aesthetic factors, such as maintaining the different colours of Finnsheep, was also addressed in the discussion. Stakeholders are active in finding alternative sources for income that utilise the native farm animal breeds [8].

### 3.3. To What Extent Are the Elements of Good Governance Met?

#### 3.3.1. Farmers’ Perspectives

According to a survey of Finnish farmers, financial incentives play a vital role in making the keeping of native breeds feasible. For the management, this study revealed several developing areas—excessive bureaucracy (57%), scarcity of subsidies (40%), and laborious requirements (37%)—as the main challenges to the subsidy system. Although it is stressed that diversity of knowledge and values are important in environmental decision making, those at the implementation level often feel that their claims and initiatives have not been realized in the planning of strategies and schemes [27].

In particular, respondents criticised the fact that the administrative protocols needed for applying the subsidies were considered rigid—everything had to be reported, often to several registers, because animal registers maintained by different parties did not communicate with one another. *“Less ‘paperwork,’ e.g., not having to spend time reporting every little thing”; “More flexibility. […] It would be really great if the subsidies could somehow be merged together, at least the requirements”*.

Of the subsidy conditions, the identification of individual animals and their replacement on a rapid schedule when needed were perceived as challenging. *“The number of animals should be sufficient, not that you need to replace individual animals”; “There should be an opportunity to report a pool of animals, so removing one would not lead to a huge paperwork, red-tape”*.

A lack of information emerged from the survey at many levels. To some respondents, it was unclear whether they were eligible for support. It also turned out that there was uncertainty with regard to the official process for managing the agreements, and other practicalities concerning the application process. Furthermore, certain conditions related to the terms of the contract, such as the identification or replacement of animals, were criticised as being too strict.

#### 3.3.2. Stakeholder Perspectives

At the stakeholder workshops, it turned out that, in the rules, there is no precisely defined timeframe for the replacement of the animal for which an owner receives a subsidy for native breeds. The lack of any clear time frames allows for a certain amount of flexibility and interpretability for the case handlers.

The stakeholders suggested, as a development measure, an option to pay the subsidy retroactively based on the actual number of animals. The second measure proposed was the development of user-friendly registration systems to improve communication between authorities. However, many stakeholders did not approve of the initiatives, and complained that they were impossible to carry out.

To summarise, the farmers were well aware of the genetic and cultural value of their native breeds. The reasons for keeping the animals were consistent with these factors. The paperwork and administrative tasks were considered too complicated and time consuming, and financial issues caused worries for the farmers. The stakeholders’ perspectives underlined the financial issues, and were mostly concerned with conservation’s biological effectiveness (i.e., maintaining the breeds’ genetic resources). Cultural aspects became more evident when discussing possibilities to diminish the dependency on agricultural support payments intended for breeds.

### 3.4. Conservation of Native Breeds in Nordic Countries

The survey was conducted among Finnish farmers keeping native breeds on their farms. However, it is possible to generalise the knowledge gained from this case study to the Nordic level and beyond when considering the similarities and differences in the conservation programmes and policies.

All Nordic countries emphasise in vivo conservation in their national strategies through their action plans [20,21,22,23,24]. The strategies define that in vivo practices should be the primary aim (Sweden), prefer in vivo over in vitro (Denmark), focus more on in vivo (Finland), and use in vitro as a complementary method (Norway). Iceland has formulated informal goals similar to those mentioned by other Nordic countries.

Nordic countries have defined operational objectives in their strategies. For example, Norway aims to expand in vitro conservation for cattle, sheep, and goats annually. According to the Danish strategy, in vitro conservation will be expanded when necessary. Only Sweden and Finland have set a formal goal in their strategies for genetic conservation as insurance against breed extinction, management of genetic diversity of in vivo populations, and research. However, Norway does have in vivo conservation of national poultry breeds.

The governance of conservation measures in Nordic countries is mainly carried out using two models: in Sweden (Swedish Board of Agriculture), Denmark (Danish Advisory Committee for the Conservation of Animal Genetic Resources), and Iceland (the Agricultural Genetic Resource Committee), the expert panel groups manage conservation activities and exercise decision-making power, while an individual organisation has the main management responsibility in Finland and Norway.

The Natural Resources Institute Finland (LUKE) is responsible for and coordinates Finland’s National Genetic Resources Programme for Agriculture, Forestry, and Fishery [21]. Similarly, in Norway, the Norwegian Genetic Resource Centre has the main responsibility for governing the programme. In all of these countries, the practical implementation of the action plan is carried out in cooperation with the respective breed associations.

Notable differences between the Nordic countries can be found, for example, in the decision making pertaining to practical measures. For example, in Sweden, breed associations are involved in selecting the materials to be stored. In Finland, the coordination of conservation programmes makes practical decisions based on their expertise under the auspices of the Advisory Committee on Genetic Resources of the Ministry of Agriculture and Forestry.

In short, regardless of the differences in governance structures, all Nordic countries highlight the importance of in vivo conservation. This implies that, in order for conservation to succeed, there must be farmers who are both willing and able to keep the native breeds. Therefore, aspects of genetic and cultural value, as well as those of good governance—including financial issues—need to be focused on.

## 4. Discussion

### 4.1. Genetic and Cultural Importance

This study indicates that half of the farmers were motivated by the conservation of genetic resources when keeping native breeds. Nevertheless, the animals’ characteristics and qualities appear to be just as important. The results also reveal the importance of financial incentives—subsidies have made it financially feasible for farmers to keep native breeds, and without them, conservation of native breeds would not be possible. In many cases, however, the amount of financial support is not enough to compensate for the gap between the higher yields of commercial breeds and the preservation of native breeds.

According to this research, farmers prefer animals that fit their production systems. A recently published report from Norway [32] shows that the number of native cattle doubled from 2012 to 2020. However, the increase in breeds is mainly from suckler cows for landscape management and meat production, while the number of native cattle in dairy production systems is relatively stable. This also reflects the general trend of cattle production in Norway.

Based on this study’s survey, the current subsidy system does not consider the production system of these animals, but only compensates for the number kept. This can lead to new governance issues regarding whether genes associated with certain traits will be lost if animals are not kept in a traditional form of production. For example, with dual-purpose cattle, will milk production capacity eventually be lost if animals are kept only for meat production? Some evidence points towards transgenerational epigenetic effects in controlling milk production (e.g., [33,34]).

It is well known that farming customs have changed dramatically throughout history. In the past, cows did not allow milking without the calf being present (e.g., [35]). It has been assumed that the tradition of keeping cattle indoors for winter and the increasing bond between humans and cows was one reason for the disappearance of this trait [36]. Considering this, changes in production systems will cause some alteration in the composition of animals’ genetic resources. Thus, adding a multidisciplinary approach to conservation measures will increase the conservation of genetic diversity before all of the necessary genetic regulatory mechanisms are sufficiently understood.

Livestock breeds illustrate human creativity, intuition, and efficiency [10]. Moreover, according to this study, cultural values are an important motivation for keeping native breeds. Respondents were also motivated by social impacts, such as intergenerational values, locality, and rarity of the breeds. Traditionally, local native breeds have played a central role in the livelihoods of the rural poor [37]. However, this study shows that this setup has been reversed: keeping the native breeds now demands finances in addition to motivation, since often their income is not high enough to cover their expenses. In a few cases, indigenous breeds produce luxury goods, which bring a better livelihood.

The term “cultural” was not strictly defined in the questionnaire, but was given to include history and tradition. In the open answers, the long-term local history of the animals was sometimes mentioned, although the answers were not detailed or abundant enough for closer analysis. History and tradition could refer to more recent historical events in the last few hundred years, or to the tradition of animal husbandry spanning thousands of years to prehistory. Regardless of the timespan, data on the human–animal relationship and the domestic animal’s cultural significance and role in livelihoods could help people to experience connection and continuum, and to be involved in preserving living history. This is likely to be a useful motivation for native-breed conservation programmes.

In his ethnographic account of Northern Finncattle and Finnsheep, Tamminen [38] showed how these animals are understood as essential to the formation of a nation, and what he calls “nonhuman citizenship”.

Respondents were also motivated by social impacts, such as intergenerational values, locality, and rarity of the breeds. Alternative incentives are aesthetic values, animals as national symbols, and human wellbeing (i.e., companionship, and affection). These results are consistent with those of previous studies (e.g., [18]).

### 4.2. Governance Aiming to Enhance the Conservation of Native Breeds

This study provides an overview of Nordic governance on the conservation of native breeds. The case study of Finnish farmers identified several obstacles hindering the conservation of native breeds. The farmers mentioned the excessive level of bureaucracy, scarcity of subsidies, and rigid application system with laborious requirements as the most significant challenges in the system. Stakeholders identified the biggest challenges as a deficiency in cooperation between authorities, lack of information, and the absence of shared registration systems. These are also obstacles to social sustainability, and are detrimental to “supporting the keepers of the genes” (a term adopted from the International Union for Conservation of Nature—a pastoralist portal), which is an essential indicator of good governance. In many cases, the results may apply to neighbouring areas.

In a recent meta-study [39], it was stressed that crucial elements of conservation include (1) cooperation among community members, and between the community and external agents; (2) social capital providing the ability to use formal and informal institutions to implement effective arrangements, making the monitoring of resource use easier to achieve; (3) leadership encouraging local empowerment and facilitating participation; (4) land tenure ensuring that the community can appropriate the benefits; and (5) adaptation providing resilience against internal and external threats.

Bennett et al. [18] contend that ecological effectiveness, social impacts, and good governance acknowledged by the local community are the most important factors for successful conservation initiatives. Greater attention to the means of conservation increases the likelihood of success. Leroy et al. [6] emphasise that the sustainable management of genetic resources depends on the participation of a diversity of stakeholders.

Efficient management of animal genetic resources also requires “good governance” for the simultaneous implementation of complementary measures. Since it also involves many stakeholders—both public and private—the definition of good governance depends on the target group concerned. In general, the stakeholders evaluate good governance based on normative principles, such as recognition, transparency, accountability, communication, participation, consultation, conflict management, trust, rule of law, legitimacy, coordination and collaboration in sharing responsibilities, and decision-making power at the local level when affecting people’s lives and livelihood [18]. Efficient results in conservation activities require close dialogue between different levels and sectors of governance. Several studies have highlighted the importance of communication between breeders and stakeholders (e.g., [16,39]) in order to (1) reach consensus on determining conservation measures, and (2) ensure that breeders are motivated to comply with the measures adopted.

From the farmers’ viewpoint, improvement of governance includes removing policy disincentives and supporting local development and livelihoods related to local animal genetic resources—similar to the issues that farmers identified in this current study. Both farmers and stakeholders identified an insufficient level of information as an issue, as was also recognised by Gicqueal et al. [40]. They also emphasised the relevance of strengthening farmers’ associations and providing practical indicator tools for managing breeding material.

The importance of tenure and livelihood security, and relations of trust, communication, and respect (i.e., elements of good governance), should not be overlooked. These aspects provide a more complex, historically and culturally contingent picture of conservation, and improve the chances of success [9]. The idea is that conservation is a place-based, context-dependent process—not a one-size-fits-all solution.

While acknowledging the importance of subsidies and other current conservation policies, we wish to underline that the production system, alongside culture and tradition, matters. Moreover, it is important to leave room for genetic material to evolve in its present-day production environment. To maintain a conservation programme in the best and most effective way, it is important to implement diverse support policies and multiple actions.

### 4.3. Towards the Multidisciplinary Future of Native-Breed Conservation Programmes?

The open-field answers in this pilot study exhibit a variety of reasons for keeping native animal breeds. However, we conclude that a more focused survey allowing statistical data collection could be planned. The aim could be, for example, to profile different groups of motivated animal keepers based on their given reasons for animal keeping, and combine these data with the impact (e.g., animal numbers, active time span, number of offspring) these groups have on the conservation program.

Furthermore, these results support the use of a multidisciplinary approach in native animal breed conservation. In our current 3MC Nordic Mountain Cattle project [26], we aim to increase awareness about the traditions and history related to Nordic Mountain Cattle breeds, and to promote the breeds themselves. In the 3MC project, this is accomplished by close cooperation with breeders’ associations, public exhibitions, and the development of a mobile game based on the cultural history of cattle in the region. However, as conservation schemes are often maintained by natural scientists and agronomists, multidisciplinary studies of native animal breeds are mainly based on separate projects. Thus, developing long-term conservation programs based more on cultural values would require more permanent input from historical and cultural studies. As the personnel resources at the national level are often limited, this work could be organized by umbrella organizations, such as NordGen, at the multinational level. The 3MC pilot project is a good example of a novel concept for cross-border collaboration in the governance of genetic material in unison with the environment, preserving traditions and cultural heritage by utilizing information gained from the past.

## 5. Conclusions

Half of the Finnish farmers were motivated by the conservation of genetic resources while keeping native breeds. The main rationalizations were animals’ characteristics, qualities, and financial incentives. Regarding various aspects of cultural values, the term was not strictly defined in the questionnaire. Despite that, respondents made reference to history and traditions. Moreover, social impacts—such as intergenerational values, locality, and rarity of breeds—were mentioned as motivators in the open answers. In order to obtain detailed information about different drivers, the terminology needs to be further defined in future studies.

This study revealed several development areas for managing conservation programmes, such as the need to reduce administrative tasks and increase subsidy levels. Farmers prefer animals suitable for their production systems; however, this is not properly supported by the current subsidy system. This raises concerns about whether genes associated with certain traits will be lost if animals are not kept in a traditional form of production. Moreover, in order to manage the conservation effectively, a comprehensive understanding of the values connected to native breeds and their genetic resources from several perspectives is needed. Based on this study, the human–animal relationship and the domestic animal’s cultural significance and role in livelihoods are useful motivations for native-breed conservation programmes. Furthermore, more dialogue between decision-makers and farmers, and opportunities to participate in decision-making processes, were desired by the “keepers of the genes”.

Our results support using a multidisciplinary approach in native animal breed conservation in order to increase awareness about the traditions and history related to animal genetic resources and their multiple values. Based on this descriptive study, a more focused survey allowing statistical data collection can be planned.

## Figures and Tables

**Table 1:** Number of farm animal breeds kept by the respondents.

Farm Animal Species	Breed	Number	%
Cattle	Eastern Finncattle	90	14.4
	Northern Finncattle	108	17.3
	Western Finncattle	117	18.7
Chicken	Finnish Landrace Chicken	34	5.4
Goat	Finngoat	13	2.1
Horse	Finnhorse	52	8.3
Sheep	Finnsheep	152	24.3
	Kainuu Grey Sheep	35	5.6
	Åland Sheep	24	3.8

## Data Availability

The data that support the findings of this study are available from the corresponding author, [M.H.], upon reasonable request.

## Appendix A

### A Webropol Questionnaire for Farmers

The questionnaire was sent to e-mail addresses in early August 2018, and a reminder was sent to all e-mail addresses in late August 2018. The main questions, with the possibility of free words, were:

(1) What native breeds do you keep?

Open answer

(2) Why do you keep native breeds?

Suggested options: genetic reasons; cultural reasons (including history and tradition); business (including speciality products and tourism); other, what? It was possible to choose several options, and to give an open answer.

(3) Have you made an environmental agreement with the government on native breeds?

Options: yes, to all breeds; yes, to some breeds; no, why? It was possible to give an open answer.

(4) What challenges do you recognise in the current support system?

Suggested options: too much bureaucracy; support payment too low; support requirements too high; other, which? It was possible to give an open answer.

(5) Has the current support system increased your willingness to keep native breeds?

Options: yes, how; no, why; I do not know. It was possible to give an open answer.

(6) How would you develop the support system?

Options: raise support, how much; ease the requirements, how; eliminate bureaucracy, how; shift the support system towards PES (payments for ecosystem services); other, what?

(7) How would you want the native breeds to be conserved?

Open answer
